# Approaches towards improving the quality of maternal and newborn health services in South Asia: challenges and opportunities for healthcare systems

**DOI:** 10.1186/s12992-018-0338-9

**Published:** 2018-02-06

**Authors:** Naeem uddin Mian, Muhammad Adeel Alvi, Mariam Zahid Malik, Sarosh Iqbal, Rubeena Zakar, Muhammad Zakria Zakar, Shehzad Hussain Awan, Faryal Shahid, Muhammad Ashraf Chaudhry, Florian Fischer

**Affiliations:** 1 Contech International, Lahore, Pakistan; 2 Institute of Social and Cultural Studies, Lahore, Pakistan; 3 Bielefeld University, School of Public Health, Bielefeld, Germany

**Keywords:** South Asia, Quality, Quality improvement, Maternal and newborn health

## Abstract

**Background:**

South Asia is experiencing a dismal state of maternal and newborn health (MNH) as the region has been falling behind in reducing the levels of maternal and neonatal mortality. Most of the efforts are focused on enhancing coverage of MNH services; however, quality remains a serious concern if the region is to achieve expected outcomes in terms of standardised MNH services within healthcare delivery systems. This research consists of a review of South Asian quality improvement (QI) approaches/interventions, specifically implemented for MNH improvement.

**Methods:**

A literature review of QI approaches/interventions was conducted using the PRISMA guidelines. Online databases, including PubMed, the Cochrane Library and Google Scholar, were searched. Primary studies published between 1998 and 2013 were considered. Studies were initially screened and selected based upon the selection criteria for data extraction. A thematic synthesis/analysis was performed to organise, group and interpret the key findings according to prominent themes.

**Results:**

Thirty studies from six South Asian countries were included in the review. Findings from these selected studies were grouped under eight broad, cross-cutting themes, which emerged from a deductive approach, representing the most commonly employed QI approaches for improving MNH services within different geographical settings. These consist of capacity building of healthcare providers on clinical quality, clinical audits and feedback, financial incentives to beneficiaries, pay-for-performance, supportive supervision, community engagement, collaborative efforts and multidimensional interventions.

**Conclusions:**

Employing and documenting QI approaches is essential in order to measure the potential of an intervention, considering its cost-effectiveness, feasibility and acceptability to communities. This research concluded that QI approaches are very diverse and cross-cutting, because they are subject to the varied requirements of regional health systems. This high level of variability leads to implementation and knowledge-management challenges for MNH programme planners and managers in the countries of the South Asia region.

**Electronic supplementary material:**

The online version of this article (10.1186/s12992-018-0338-9) contains supplementary material, which is available to authorized users.

## Background

South Asian countries face huge public health challenges, particularly in maternal and newborn health (MNH) [1]. This is evident from the high infant mortality rates of India, Pakistan, Afghanistan, Bangladesh and Nepal [2, 3]. Hence, quality improvement (QI) in MNH care is a serious concern. It is evident that the process of QI requires sound local strategies in order to attain the best possible results by focusing on the need to optimise resource use and expand population coverage. It follows from this that increased expertise and resources will not, in themselves, translate into MNH care of high quality. This observation is supported by the fact that countries with the highest health spending are not always those with the highest quality healthcare system [4]. Quality improvement can be termed as a multidimensional concept, which draws upon several perspectives [5–7]. Initially, QI mainly concentrated on biomedical outcomes, however, over time its conceptualisation broadened and implied different meanings for varied stakeholders [7]. Patients view effectiveness of services, empathy, clean and safe environment and facilities as indicators of QI with special focus on readily access to the experienced and helpful provider, whereas healthcare providers regard efficacy of treatment, quality of care according to the clinical guideline as key QI intervention. Managers, on the other hand, emphasise client satisfaction and resource utilisation, while for policy makers, preference to access, cost, equity and effectiveness are the most critical attributes of quality improvement [7]. Thus, QI acknowledges the notion that quality must focus on optimum resource utilisation, using sound local strategies to address components pertaining to service users, patient satisfaction, sustainability and other aspects of healthcare [8, 9].

Over the past few decades, there has been a growing awareness of the need to improve quality across healthcare delivery, driven by the need to reduce inequalities and effectively translate evidence into practice alongside the changing expectations of patients and care-givers [10]. QI approaches to healthcare have been researched internationally; nevertheless, there is limited documentary evidence at a regional level in South Asia. For this reason, we carried out a review to document the various regional approaches and interventions, implemented to bring quality improvement in MNH services, particularly focusing on the achievement of the Sustainable Development Goal 3. The proposed research question for this review is to explore which key interventions/approaches have been applied for bringing QI in MNH services within the South Asian region.

## Materials and methods

We adopted the quality assessment tool ‘PRISMA’, developed by the World Health Organisation (WHO), to perform the literature review [11] (Additional file 1).

### Eligibility

We identified studies published between 1998 and 2013 in indexed national and international journals in the English language, focusing on QI approaches/interventions designed to improve MNH services in the regional countries of South Asia. Quality improvement interventions/approaches were defined as any systematic processes or actions aimed at reducing the quality gaps and leading to measurable improvements in MNH services and health status of the targeted populations [12, 13].

### Identification of studies

We reviewed literature from academic databases, as well as project reports and documents. Key sources of electronic searches included reference libraries; namely, PubMed and the Cochrane Reference Libraries, along with Google Scholar. The goal was to access the available data on QI approaches for improving MNH services. While searching the databases, we employed various medical subject heading (MeSH) search terms, such as ‘maternal health, neonatal/newborn health, health services, and maternal welfare’. Other key terms included ‘healthcare performance, interventions/approaches and quality improvement’. Our search strategies were then refined through the adoption of an iterative approach. In addition, a manual search was undertaken, consisting of the detailed examination of cross references and the bibliographies of selected publications to identify additional sources of information. The search was further widened to include a review of the grey literature of international and national organisations as well as development partners. The search comprised of both experimental and observational studies.

### Study selection

Initially, we screened all the studies’ titles and abstracts and repeated this process to ensure consistency. Studies were included in the review, which met one of the following criteria: 1) QI approaches/interventions with a primary focus on improving MNH services in South East Asian countries (including antenatal care, delivery care, postnatal care, neonatal care at birth, neonatal emergency care and neonatal primary care); or 2) QI approaches/interventions targeted at stakeholders including healthcare providers, health managers and beneficiaries for improving the MNH status of poor/marginalised communities in the region. Furthermore, only those selected studies as per the above criteria were reviewed, where QI approaches/interventions have the potential for scaling-up. We excluded QI approaches that were not tested within the countries of South Asia.

### Data extraction

We extracted data from all the studies identified, as fulfilling the above criteria. Two researchers independently reviewed the full text of these papers. A spreadsheet was used for data extraction. Finally, both researchers critically reviewed all the selected studies and resolved any observed inconsistencies through discussion. The data was extracted using the following parameters: 1) geographical setting and target population; 2) description of QI approaches/interventions and relevant set of services provided; 3) outcome; and 4) key findings, highlighting improved MNH services.

### Synthesis of results

We adopted an analytical approach of thematic synthesis, in which data from selected studies was categorised, grouped and interpreted according to the prominent themes, with the aim of identifying common elements across the studies [14]. The analysis began with a deductive approach through which emerging themes and sub-themes were initially identified. All QI approaches/interventions implemented to improve MNH services were analysed, considering their quality measures, the strategies adopted, the main results and outcomes. Furthermore, they were grouped together to highlight key themes and summarised in tabular form (Additional file 2).

## Results

**Fig. 1:**
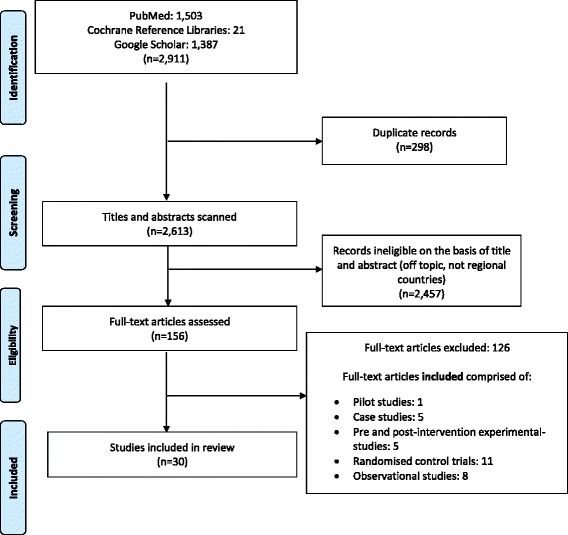
PRISMA flow chart for study selection

The electronic database search yielded a large number of studies, which reflected the widespread discussion on QI approaches for improving MNH services. A total of 2613 unique records were retrieved, out of which 156 full texts were screened. Finally, 30 studies on QI approaches/interventions were selected for analysis from six South Asian countries: Afghanistan, Bangladesh, India, Nepal, Pakistan and Sri Lanka. In Fig. 1, the flow of studies through searching and screening for inclusion is described.

### Quality improvement approaches/interventions

The analysis of selected studies highlighted eight broad cross-cutting themes as the most commonly applied QI approaches/interventions for improving quality of care in the South Asian region. Nine studies focused building the capacities of healthcare providers in clinical quality, two in clinical audits and feedback, two discussed providing financial incentives, one each highlighted improvements in health workers’ performance, efficiency through pay-for-performance schemes and supportive supervision, eight studies dealt with community engagement, four elucidated collaborative efforts or public/private partnerships and the remaining two addressed multidimensional interventions.

#### A) Capacity building of healthcare providers in clinical quality

Capacity building of healthcare providers (HCPs) on improvements in quality of care, knowledge level, adherence to clinical protocols and changes in clinical practice behaviour was adopted as a proven QI approach to improve the quality of MNH services. The results indicated that most of the studies focused on the capacity building of Traditional Birth Attendants (TBAs) for delivery assistance, particularly in the use of delivery kits, enhanced referrals and linkage with community health workers as a key QI approach. Evidence from Pakistan showed a 31% reduction in stillbirths, 29% in the neonatal mortality rate (NMR) and 39% in haemorrhage-related complications during pregnancy, along with a 50% increase in referrals for emergency obstetric care, after deploying TBAs in communities for perinatal care [15, 16]. Similarly, an investigation into the training of TBAs on clean delivery kits revealed that trained TBAs were twice as likely to perform a clean delivery in rural Bangladesh. However, evidence was lacking to support the positive effect of training TBAs in the occurrence of maternal infections [17]. The findings revealed that training HCPs in antenatal care (ANC), routine visits, regular intake of vitamin supplements, recognition of danger signs, and measures for intermittent preventive treatment during pregnancy and postnatal care showed comparatively better results in utilisation of MCH services. Furthermore, after building capacity on the integrated management of childhood illnesses (IMCI), a significant reduction was observed in the incidences of malaria, pneumonia and diarrhoea amongst children in comparison to other areas [18].

Multiple studies showed that training in emergency newborn care has improved newborn health outcomes in regional countries like Sri Lanka, Bangladesh, India and Pakistan. Various QI approaches/interventions were applied to promote the practices of thermal protection, preventing asphyxia, preparedness for resuscitation and neonatal assessment, along with the management of low birth weight, respiratory distress, feeding and neonatal sepsis [19–22].

#### B) Clinical audits and feedback

Clinical audit and feedback is a proven QI approach, involving the systematic critical analysis of the quality of clinical care given to patients, focusing on such factors as procedures used for diagnosis and treatment, use of resources and outcome [23]. Our findings indicated that audits of clinical practice, maternal audits and the development and implementation of guidelines have added value to clinical performance, service utilisation and patient satisfaction [24]. Moreover, the use of quality scorecards at health facilities and their dissemination to communities were some of the diverse methods implemented for improving the quality of clinical services [25].

#### C) Financial incentives for beneficiaries

The provision of free-of-cost services or financial incentives for beneficiaries as QI approaches/interventions were found to be successful strategies, resulting in improved service utilisation and user satisfaction. A study conducted in Nepal examined the provision of free maternity care through reimbursement to facilities from government funds for facility-based deliveries. The results showed a substantial increase in facility-based deliveries, management of complications and caesarean sections. Furthermore, beneficiaries experienced minimal delays in receiving care at these facilities [26]. Meanwhile, maternal health voucher programmes specifically targeted at safe deliveries have expanded in Bangladesh, India and Nepal [27]. These vouchers increased the number of deliveries taking place at health facilities [28]. Social protection mechanisms in the form of insurance cover for obstetric risks implemented in India increased the average number of ANC visits and ultrasound examinations during ANC visits [18].

#### D) Pay-for-performance

Improving the performance of HCPs through pay-for-performance is a continuous QI approach designed to deliver the best services. Providing performance-based payments to HCPs under the Accredited Social Health Activists (ASHAs) programme in India led to an increase in both facility-based deliveries and breastfeeding practices, as well as a decline in neonatal mortality of up to 70% [29, 30]. Moreover, in Pakistan under the Chief Minister’s initiative for the attainment and realisation of MDGs (CHARM), all staff members, from medical officers to drivers and security guards, were incentivised to encourage facility-based deliveries [31].

#### E) Supportive supervision

Supportive supervision is a facilitative approach of supervision, providing necessary leadership and support for QI and emphasizing mentorship, joint problem solving and two-way communication between supervisors and those being supervised [32]. Supportive supervision is an optimal QI approach for clinical settings as it overturns traditional notions of supervision and focuses on facilitation rather than inspection [32]. Overall, supportive supervision of HCPs within the health system demonstrated better MNH outcomes and augmented motivation. This is evident in an example of supervisory strategy adopted by the Bangladesh Rural Advancement Committee (BRAC) Shasthya Shebikas, in which strong supervision was provided for community health workers (CHWs) from other CHWs with a higher level of training (Shasthya Kormis). Unlike traditional health professionals, these supervisors were found to be fully capable of performing their duties within their community and improving MNH outcomes [33–35].

#### F) Community engagement

Studies highlighting community engagement mainly focused on mothers’ education in birth preparedness, the optimum number of ANC visits, recognition of danger signs and newborn care. The results showed a raised awareness and increased utilisation of MNH services in countries in the region [36, 37]. However, no significant effect on mortality was observed, except in a few areas of Bangladesh where neonatal mortality significantly decreased with the introduction of these practices and the resulting enhanced knowledge about maternal and neonatal danger signs [38]. A comprehensive process of participatory learning and an action cycle also implemented in Bangladesh showed no effect on maternal or newborn mortality [39]. Another example of community engagement included regular visits by trained midwives to the community for counselling, which significantly increased the initiation of breastfeeding in Bangladesh and contraceptive uptake by women in Pakistan and Nepal [40–42]. Moreover, behavioural change communication addressing lactation and contraception in rural areas of India not only increased knowledge of the lactational amenorrhea method but also promoted birth spacing [43].

#### G) Collaborative efforts/contracting out

Collaborative efforts included public/private partnerships (PPPs): contracting out arrangements between government and private entities for the provision of MNH services, such as infrastructure, equipment, human resources or technical health services [44]. The findings indicated that delays in treating obstetric emergencies leading to maternal mortality were reduced in rural areas of Pakistan and Bangladesh with the provision of ambulances, the meeting of associated expenses and a referral system [44, 45]. An example from Uttar Pradesh, India, highlighted the provision of contraceptives at subsidised rates for distribution and sale to private providers, which increased the availability of contraceptives in the state [46]. Similarly, the government of Afghanistan, in collaboration with international partners and government and non-government organisations, designed a Basic Package of Health Services (BPHS) for the provision of MNH services to its rural population. Following this intervention, BPHS facilities increased from 1075 in 2004 to 1829 in 2011. Thus, there was an overall increase in attended births [47].

#### H) Multidimensional interventions

Multidimensional QI approaches included those studies considering a combination of two or more strategies, implemented within large-scale programmes aimed at improving MNH outcomes. Facility upgrading (infrastructure, equipment, drugs and supplies) was seen as the most common QI approach, coupled with other integrated approaches, such as staff training, monitoring and supervision, strengthening of the referral system, the use of audits and guidelines, contracting out, improved data management and logistic support [48]. For example, the quality of ANC services was increased in rural India by ensuring the provision of necessary equipment, focused training and the reorganisation of outreach services. The findings showed that the delivery of special preventive packages comprising essential newborn care (ENC) service workers resulted in improving birth preparedness and delivery outcomes, with enhanced breastfeeding practices in Shivgarh India [49].

## Discussion

This review aimed to identify QI approaches/interventions with proven effects on upgrading the MNH services implemented in countries of the South Asian region. QI approaches in healthcare settings usually target one or more of the following three groups: health managers, healthcare providers and end-users/beneficiaries. This review considered all of these perspectives, while primarily focusing on improved MNH outcomes from the beneficiaries’ point of view, which is still rarely considered in many settings [50].

The overall findings revealed mixed evidence, involving various cross-cutting QI approaches being employed to improve MNH services with varying level of success. It revealed that the most effective QI approaches are carefully rationalised, considering the needs of the target population, the socio-cultural context and the quality of healthcare services. Moreover, the knowledge and motivation of healthcare providers, as well as their interactions with clients and supportive supervision, also boost their performance.

Capacity building of HCPs in the area of clinical quality was found to be the most common QI approach within low-resource settings, in contrast to other QI approaches where extensive financial inputs are involved. Regional evidence revealed that strengthening the capacity of HCPs, particularly TBAs and CHWs, is an effective approach to reducing MNH morbidity and mortality [51, 52]. The situation is similar for countries such as Pakistan, India and Bangladesh, where most deliveries are conducted by TBAs, who require continuous support in the form of training and referral to save mothers and children [53, 54].

The studies showed that clinical audits and feedback mechanisms have a significant effect on case fatality rates and maternal mortality. They also indicated that the implementation of referral guidelines [23] and the dissemination of performance indicators via scorecards [24] incorporated elements of an evaluation-action cycle for developing strategies for QI. The improvements outlined were significantly associated with participatory methodologies involving service providers and facility management [25].

The provision of financial incentives and supportive supervision was significantly associated with improved MNH outcomes. Regional evidence suggests that monetary incentives for beneficiaries and rewarding HCPs for better performance both lead to an improvement in MNH outcomes [26, 27]. Quality of care was reported as a concern of the poor and disadvantaged. Therefore, strategies centred around reducing out-of-pocket expenditure through targeted subsidies and risk insurance leads to improvements in service uptake by the poorest quintile [36, 37]. Moreover, the upgrading of facilities through improvements in infrastructure, equipment, drugs and supplies was also seen as a significant QI approach, in areas where sufficient resources and financial commitment is available [31, 32]. Such findings have significant linkages with the situation of low-middle-income countries in South Asia as facility readiness in this region was documented to be far below acceptable levels, particularly at public facilities [54].

It seems that service uptake tends to increase when strategies are mainly focused on patient perceptions. Interventions such as awareness-raising sessions led to an increased utilisation of ANC services and greater client satisfaction, and meant that patients were more likely to refer new women to the facility than those receiving care from TBAs [35, 52]. Furthermore, assigning a designated service provider during the entire process of pregnancy and the postnatal period increased the utilisation of services and improved continuity of care [36–38]. Exposure to contraceptive counselling and educational visits by trained midwives not only led to a significant increase in contraceptive knowledge and uptake, but also resulted in the more frequent practice of birth spacing [39–41]. Collaborative efforts aimed at health education and the provision of medical supplies at subsidised rates went on to produce desirable results [44].

### Limitations

The majority of studies included in this review were based on intervention impact assessments rather than surveys or interviews, which adds to its consistency. However, this paper only highlights the different interventions and their outcomes without assigning any weights to the effectiveness of each implementation. Certain significant studies in further languages were not incorporated into the data synthesis, considering the review was limited to three databases and English language only. This may be considered a substantial limitation of this review. In addition, some significant studies with regional representation could not be included due to open-access issues, particularly for Bhutan and the Maldives.

## Conclusion

The QI approach is key to improving equity in MNH service delivery. Our research concluded that QI approaches are diverse and cross-cutting and subject to the varied requirements of regional health systems. This review reflects that there is a high level of variability of QI approaches, subject to the selection of any QI intervention, considering the varied needs of stakeholders within particular geographical context, leading to implementation and knowledge management challenges for MNH programme planners and managers in countries within the region.

Employing and documenting QI approaches is essential in order to measure the potential of each intervention, considering its cost-effectiveness, feasibility and acceptability to communities. Summarising the above, the process of quality improvement does not end with implementation. It requires extensive and continuous efforts, resources and technical expertise. Ultimately, QI approaches to improving MNH services are only viable if they are sustainable and integrated into the health systems, paving the way to attaining sustainable development goals in the region, particularly for improving health status, promoting well-being and reducing maternal and newborn mortality.

## Additional files

Additional file 1: PRISMA 2009 Checklist. (DOC 66 kb)

Additional file 2: Description of reviewed studies disaggregated by QI interventions. (DOCX 39 kb)

## Abbreviations

ANC: Antenatal care
ASHA: Accredited Social Health Activists
BPHS: Basic Package of Health Services
BRAC: Bangladesh Rural Advancement Committee
CHARM: Chief Ministers initiative for the attainment and realization of MDGs
CHWs: Community health workers
EmOC: Emergency obstetric care
HCPs: Health care providers
IMCI: Integrated management of childhood illness
MeSH: Medical subject headings
MNH: Maternal and newborn health
NMR: Neonatal mortality rate
PNC: Post-natal care
PPP: Public private partnerships
QI: Quality improvement
TBAs: Traditional Birth Attendants
WHO: World Health Organization
