# Supplementary material for: Approaches towards improving the quality of maternal and newborn health services in South Asia: challenges and opportunities for healthcare systems
Source: Global Health. 2018 Feb 6;14:17. doi: 10.1186/s12992-018-0338-9 (PMC5802097; doi:10.1186/s12992-018-0338-9)
Supplement: Supplementary file 2 — Description of reviewed studies disaggregated by QI interventions. (DOCX 39 kb) [file 12992_2018_338_MOESM2_ESM.docx]

**Additional file 2: Description of reviewed studies disaggregated by QI interventions**

| **Study author & year** | **Geographical setting** | **Type of research** | **QI intervention** | **Outcomes** | **Key findings** |
| --- | --- | --- | --- | --- | --- |
| Capacity building of healthcare providers (HCPs) on clinical quality | | | | | |
| 1. Jokhio et al., 2005 | Rural Pakistan | Clustered randomised controlled trial | Training of TBAs for enhanced referrals, ANC and PNC visits, and provision of delivery kits. TBAs were also linked with community-based Lady Health Workers (LHWs). | Reduction in pre-term mortality, still births, neonatal mortality and pregnancy related complications | Intervention did not show any impact on maternal mortality, however Preterm Mortality reduced by 30%, still births reduced by 31%, NMR reduced by 29%, 39% reduction in hemorrhage related complication during pregnancy and 50% increase in referrals in emergency obstetric care. |
| 1. Bhutta et al., 2008 | Rural Pakistan | Pilot study | TBAs linked with community based Lady Health Workers (LHWs). The LHWs in intervention clusters received additional training focused on essential maternal and newborn care and conducted community education group sessions | Reduction in neonatal mortality and still birth rate | The average stillbirth rate decreased from 65.9 to 43.1 per 1000 birth, while the neonatal mortality rate decreased from 57.3 to 41.3 per 1,000 live births. |
| 1. Goodburn et al., 2000 | Rural Bangladesh | Observational | Training of TBAs in clean delivery | Increased clean delivery practices | Trained TBAs were twice as likely as untrained TBAs to perform clean delivery (45% vs. 19.3%); however, no significant differences in regards to maternal infection. No association between maternal infection and clean delivery. Maternal infection significantly associated with existing RTI, insertion of hand into vagina, malnutrition and non-primagravidity. |
| 1. Barua et al., 2003 | Rural District, Maharashtra State, India | Observational | ANC improvements - ensuring staff had necessary equipment, increased monitoring and supervision, focused training, re-organisation of outreach clinics and scheduling. | Improvement in quality of care | Quality of care increased, >60% of women availed all ANC components. Blood pressure and urine testing increased significantly (from 50 to 94% for BP and 46-89% for urine). Attendance at clinics rose by 30% (from average for 4-5 women per camp to 15-20 women per camp) |
| 1. Senarath et al., 2007 | Sri Lanka | Randomised controlled trial | Training in essential neonatal care | Improvement in newborn care and practices | Newborn practices showed significant improvement for cleanliness at delivery, thermal protection, preparedness for resuscitation and neonatal assessment. Breastfeeding initiation within 30 minutes showed significant increase (45.3% to 70.4%). |
| 1. Arifeen et al., 2009 | Rural Bangladesh | Cluster randomised controlled trial | IMCI with training, supervisory support, supply tracking system, basic job aids and referral guidelines | Improvement in health service provision and reduction in under 5 mortality | Significant improvement was seen in mean index of correct treatment and counselling in IMCI facilities from 8% to 54% during initial periods of the program.  Under 5 mortality rates fell from 70.0 and 65.6 per 1,000 live births in intervention and comparison areas to 49.3 and 50.5 per 1,000 live births. Significant accelerations in reductions of mortality were also observed. |
| 1. Tholpadi et al., 2000 | Rural India | Pre and post-intervention experimental study | Neonatal resuscitation training of village health centre physicians, nurses, birth attendants; use of performance checklist; refresher in 3 months | Improvement in quality of care | Training improved management of asphyxia after organisation of trainings |
| 1. Mufti et al., 2006 | Teaching Hospital in Pakistan | Pre and post-intervention experimental study | Training of health care providers in management of low birth weight, respiratory distress, feeding, neonatal sepsis, and neonatal resuscitation. | Reduction in perinatal and neonatal mortality | Trainings on emergency newborn care showed improvement in perinatal and neonatal mortality. |
| 1. Arifeen, 2009 | 20 Primary Health facilities, Matlab Subdistrict, Bangladesh | Cluster randomised controlled trial | IMCI with training, additional supervision, supply tracking system, basic job aids (scales, thermometer etc.), referral guidelines were implemented | Reduction in under 5 mortality, stunting and wasting  Improvement in health service provision and utilization | Mean index of correct treatment and counselling in IMCI facilities raised from 8% to 54% between 2001 and 2004. Levels remained between 64-68% thereafter. Did not improve significantly in control. Proportion of ill children taken to appropriate provider increased in IMCI areas from 9% in 2000 to 24% in 2007, compared to controls where it remained between 4-8%.  Under 5 mortality rates fell from 70.0 and 65.6 per 1000 live births in intervention and comparison areas to 49.3 and 50.5 per 1000 live births. After adjustment for baseline rates, the difference corresponded to 3.3 fewer deaths per 1000 live births in IMCI areas. No evidence of significant accelerations in reduction of mortality. 12% decrease in stunting in children aged 24–59 months in intervention areas compared to 5% in comparison areas. Wasting dropped substantially in both areas, but differences between areas were not significant. |
| Clinical audits and feedbacks | | | | | |
| 1. Chowdhury et al., 2008 | Rural Bangladesh | Observational | Audits of clinical practices, maternal audits and implementation guidelines | Improvement in quality of care  Reduction in mortality | Appropriate management of severe pneumonia cases increased from 36% at baseline to 90% after revision of referral guidelines. Case fatality rates did not significantly change (1.1% to 0.6%). All-cause mortality significantly declined by 41% but not for pneumonia-related deaths. |
| 1. Khan et al., 2012 | Bangladesh | Observational | Use of a Balanced Scorecard in strengthening health system | Improvement in health facility performance and clients’ satisfaction | Improved feedback was recorded related to medical staff, instruments and drugs and the overall performance of the health facility. Overall, the job satisfaction was found lower amongst physicians and nurses, however, patient satisfaction score was high. |

| Study author & year | Geographical setting | Type of research | QI intervention | Outcomes | Key findings |
| --- | --- | --- | --- | --- | --- |
| Financial incentives to beneficiaries | | | | | |
| 1. Witter et al., 2011 | Nepal | Pre and post-intervention experimental study | Free maternity care services and reimbursement of facilities with Government funds for facility-based births | Improvement in Service Utilisation | Facility-based deliveries for normal births increased by 19%, for complications by 15%, and for caesarean by 18%. Clients perceived reduced delays in receiving care; however, patients were charged fees at some public facilities due to loss of revenue. |
| 1. Eichler et al., 2013 | India, Bangladesh, Nepal, Afghanistan | Observational | Performance Based Incentives to manage health services | Improved Service Provision and Service Utilisation | Supply-side performance-based incentives are associated with increased numbers of institutional deliveries. A range of individuals received performance-based payments, including the eligible women who delivered at a health facility; community health workers who accompanied the women; and also to the providers performing the delivery. |
| Pay for Performance | | | | | |
| 1. Paul et al., 2011 | India | Observational | Providing performance based payments to health care workers | Reduction in neonatal mortality | Increased both facility based deliveries and breastfeeding practices, resulting in 70% decline in neonatal mortality. |
| 1. UNICEF, 2013 | Punjab, Pakistan | Case studies | Implementation of various innovations (financial flexibility, robust monitoring and evaluation, pay-for-performance, reporting of results, and raising community involvement/awareness via mobile phone text messaging service). | Increase in service utilisation and improved health service provision | The combination of supply-side inputs with maternity health components proved to be effective in mHealth service delivery at a low cost. Pay for performance initiative incentivised provision of 24/7 health services. |
| Supportive Supervision | | | | | |
| 1. Ahmed, 2008 | Bangladesh | Observational | Assigning community health workers with a higher level of training (Shasthya Sebika) to deliver essential healthcare | Increased performance | Increased motivation and retention of Health Workers at the grassroots level in rural areas and expansion in primary health care infrastructure |
| Community Engagement | | | | | |
| 1. Dongre et al., 2009 | Rural India | Pre and post-intervention experimental study | Educate women about newborn danger signs, birth preparedness, health care seeking, and conduction of monthly village based meeting. | Improved community engagement and awareness | Significant improvement was seen in health seeking practices particularly for sick newborns |
| 1. McPherson et al., 2006 | Nepal | Pre and post-intervention experimental study | Birth preparedness plan, keychain containing information on antenatal, care of mother and newborn, danger signs | Improved community engagement and awareness | Essential newborn care preparedness increased from 20% to 30%. However, no improvement in early initiation of breastfeeding or skilled birth attendants at delivery was observed. |
| 1. Fottrell et al., 2013 | Rural Bangladesh | Randomised controlled trial | Women's groups at a coverage of 1 per 309 population that proceed through a participatory learning and action cycle in which they prioritize issues. This affected maternal and neonatal health and design and implement strategies to address these issues. | Reduction in neonatal mortality and improvement in service utilisation | The neonatal mortality rate was significantly lower in the intervention area, i.e. 21.3 neonatal deaths per 1000 live births, while 30.1 per 1000 in control areas. Reduction in neonatal mortality of 38% was observed. Improvements were seen in hygienic home delivery practices, newborn thermal care, and breastfeeding practices. |
| 1. Darmstadt et al. 2010 | Bangladesh | Randomised controlled trial | Community health workers identified pregnant women; conducted two antenatal home visits to promote birth and newborn care preparedness; conducted four postnatal home visits to negotiate preventive care practices and to assess newborns for illness; and referred sick neonates to a hospital and facilitated compliance | Increased health service provision | ANC and PNC coverage increased. Indicators of healthcare practices and knowledge of maternal and neonatal danger signs improved. Adjusted mortality hazard ratio in the intervention area was 1.02 at baseline and 0.87 at endline, as compared to the control area. |
| 1. Azad et al., 2010 | Rural Bangladesh | Clustered randomised controlled trial | Implemented a participatory learning and action cycle in which women support groups were formed to identify and prioritise problems, formulate strategies implement, monitor and finally evaluate the process. | Reduction in neonatal mortality | The effect of participatory women's groups was found to be significant on neonatal mortality. Cluster-level mean NMR was 33.9 deaths per 1000 livebirths in the intervention clusters as compared to 36.5 per 1000 in the control clusters. |
| 1. [Saeed et al., 2008](#_ENREF_2) | Pakistan | Randomised controlled trial | Exposure to contraceptive counselling and educational leaflets on contraceptive practices of couples during postnatal | Improvement in service utilisation | There was a significant increase in contraceptive uptake in women provided with educational leaflets and counselling session toward use of more reliable contraceptive methods. |
| 1. Bolam et al., 1998 | Nepal | Randomised controlled trial | One to one health education at birth and three months later were provided through midwives and community health worker. Impact of postnatal health education for mothers on infant care and postnatal family planning practices was evaluated | Improvement in service utilisation | Improvement was seen in uptake of family planning at six months after birth. However, no significant impact on maternal knowledge and practices of infant care. |
| 1. Sebastian et al. 2012 | Rural India | Randomised controlled trial | A behaviour change communication intervention integrated into the existing government program to increase knowledge and use of the lactational amenorrhea method and postpartum contraception through counselling of community health workers. | Improvement in service  utilisation | There was an increase in knowledge of the lactational amenorrhea method and use of spacing methods. Use of modern contraceptives for spacing at nine months postpartum was 57% in the intervention group as compared to 30% in the control group. |
| Collaborative Efforts/Contracting Out | | | | | |
| 1. Tech, 2009 | Uttar Pradesh, Uttarakhand and Jharkhand, India | Case studies | Public Private Partnership between SIFSPA and Hindustan Latex Ltd, Population Services, International (PSI), DKT International and Hindustan Latex Family, Planning Promotion Trust (HLFPPT) was formed for provision of contraceptives at subsidized rates for distribution and sales. | Improvement in coverage, accessibility and use of FP Services | Public Private Partnership improved the penetration and visibility of contraceptives in the state. Increased coverage, accessibility and use of IUD services through Social franchises. |
| 1. PAIMAN, 2006 | Bangladesh | Case studies | Public private collaboration between Bangladesh District Government and NGO was formed for provision of ambulances (fuel, maintenance and driver) to transport patient to hospitals to strengthen emergencies obstetric and neonatal services. | Reduction in maternal and child mortality | Emergency obstetric and neonatal services were strengthened, a decrease was seen in maternal and child mortality. |
| 1. Midhet, 1998 | Balochistan, Pakistan | Case studies | Balochistan Safe Motherhood Initiative (BSMI) was a Public Private Partnership arrangement between Government of Balochistan Health Department, UNICEF and Asia Foundation. Through this PPP, Information, Education and Communication strategies developed for women, families and traditional birth attendants; Streamlined local transport system in need of emergencies obstetric care; Introduced reliable communication system to connect traditional birth attendants and primary health facilities and transport system; Upgraded and strengthened reproductive health services and trained health care providers, including Lady Health Workers | Improvement in knowledge and service utilisation | Baseline and follow up surveys showed significant improvement in women’s knowledge and perception of reproductive health problems as well service utilisation. |
| 1. Kaartinen & Diwan, 2002 | Kabul, Afghanistan | Case studies | Basic Package of Health Services (BPHS) designed to provide maternal and child health services to rural population by collaborating with CHWs. | Improvement in service utilisation | Trained midwives were preferred for deliveries rather than traditional birth attendants within BPHS facilities for deliveries. |
| Multidimensional Interventions | | | | | |
| 1. Kumar et al., 2008 | Shivgarh, Uttar Pradesh, India | Randomised controlled trial | Provided a preventive package within intervention areas for essential newborn care (birth preparedness, clean delivery and cord care, thermal care), breastfeeding promotion, and danger sign recognition.  Community health workers delivered the packages via collective meetings and two antenatal and two postnatal household visits. | Improvement in quality of care and reduction in neonatal Mortality | Improvements in birth preparedness, hygienic delivery, thermal care (including skin-to-skin care), umbilical cord care, skin care, and breastfeeding were seen in intervention area. There was little change in care-seeking. Compared with controls, neonatal mortality rate was reduced by 54% in the essential newborn-care intervention. |
| 1. Rana et al., 2007 | 4 rural districts, Nepal | Observational | Upgraded 8 existing public health facilities through Infrastructure, equipment, training and community information activities | Facility upgradation and  Improvement in Service Provision and Utilisation | Three comprehensive and four emergency obstetric care (EmOC) facilities established in those areas where EmOC services were previously deficient. EmOC services improved from 1.9 to 16.9%, proportion of births in EmOC project facilities increased from 3.8 to 8.3% and case fatality rate declined from 2.7 to 0.3%. |
